# LyGDI, a Novel SHIP-Interacting Protein, Is a Negative Regulator of FcγR-Mediated Phagocytosis

**DOI:** 10.1371/journal.pone.0021175

**Published:** 2011-06-14

**Authors:** Payal Mehta, Anne-Sophie Wavreille, Steven E. Justiniano, Rachel L. Marsh, Jianhua Yu, Richard W. Burry, David Jarjoura, Timothy Eubank, Michael A. Caligiuri, Jonathan P. Butchar, Susheela Tridandapani

**Affiliations:** 1 The Ohio State Biochemistry Program, The Ohio State University, Columbus, Ohio, United States of America; 2 Department of Internal Medicine, The Ohio State University, Columbus, Ohio, United States of America; 3 Department of Molecular Virology, Immunology and Medical Genetics, The Ohio State University, Columbus, Ohio, United States of America; 4 Campus Microscopy and Imaging Facility, The Ohio State University, Columbus, Ohio, United States of America; 5 Center for Biostatistics, The Ohio State University, Columbus, Ohio, United States of America; 6 Comprehensive Cancer Center, The Ohio State University, Columbus, Ohio, United States of America; University of Birmingham, United Kingdom

## Abstract

SHIP and SHIP-2 are inositol phosphatases that regulate FcγR-mediated phagocytosis through catalytic as well as non-catalytic mechanisms. In this study we have used two-dimensional fluorescence difference gel electrophoresis (DIGE) analysis to identify downstream signaling proteins that uniquely associate with SHIP or SHIP-2 upon FcγR clustering in human monocytes. We identified LyGDI as a binding partner of SHIP, associating inducibly with the SHIP/Grb2/Shc complex. Immunodepletion and competition experiments with recombinant SHIP domains revealed that Grb2 and the proline-rich domain of SHIP were necessary for SHIP-LyGDI association. Functional studies in primary human monocytes showed that LyGDI sequesters Rac in the cytosol, preventing it from localizing to the membrane. Consistent with this, suppression of LyGDI expression resulted in significantly enhanced FcγR-mediated phagocytosis.

## Introduction

Fcγ receptor (FcγR) clustering on monocytes and macrophages results in the activation of a number of signaling pathways and culminates in phagocytosis [1]. This process is accompanied by the release of pro-inflammatory cytokines and reactive oxygen species that, although required for optimal clearance of immune complex (IC), will lead to tissue damage if not tightly regulated. FcγR activity and phagocytosis are governed by phosphatases such as the SH2 (Src Homology 2) domain-containing inositol phosphatases SHIP and SHIP-2 [2], [3]. These phosphatases share a high degree of homology within the catalytic domains. However, the non-catalytic domains that mediate interactions with downstream signaling proteins are largely divergent [4], [5]. Thu SHIP and SHIP-2 can associate with non-overlapping cytoplasmic signaling molecules via their non-catalytic domains and these associations may influence multiple signaling pathways. Little is known about the molecular details of these associations and their effects on ensuing biologic responses. Here we have used a highly sensitive proteomics-based DIGE (two-dimensional fluorescence difference in gel electrophoresis) approach to identify undiscovered binding partners of SHIP and SHIP-2, and found that LyGDI associates with SHIP.

LyGDI (RhoGDIβ, RhoGDI2, D4-GDI or GDID4) belongs to the family of Rho guanidine dissociation inhibitors (RhoGDI) that regulate the activity of RhoGTPases by stabilizing their cytosolic GDP-bound inactive form [6], [7]. RhoGDI accommodates the C-terminal isoprenyl motif of RhoGTPases within a hydrophobic pocket thus sequestering them within the cytosol. Following dissociation from RhoGDI and GDP to GTP exchange, RhoGTPases are translocated to the plasma membrane via C-terminal isoprenyl motif, where they activate their downstream effectors such as PAK and WASP [8]. Putative substrates of LyGDI include the RhoGTPases RhoA, Cdc42 and Rac, as suggested by *in vitro* studies using recombinant LyGDI [9]. Rac plays an important role in mediating actin cytoskeletal changes during processes such as phagocytosis and cell locomotion. Consistent with this, overexpression of LyGDI results in disruption of the actin cytoskeleton [10].

Here, we report that SHIP associates with LyGDI downstream of FcγR clustering in human monocytes. This association is indirect and requires Grb2 as well as the C-terminal proline-rich domain (PRD) of SHIP. Subsequent experiments showed that LyGDI is a suppressor of Rac membrane localization in human monocytes and that it negatively regulates FcγR-mediated phagocytosis.

## Results

### SHIP and SHIP-2 associate with distinct signaling intermediates upon FcγR clustering

**Figure 1:**
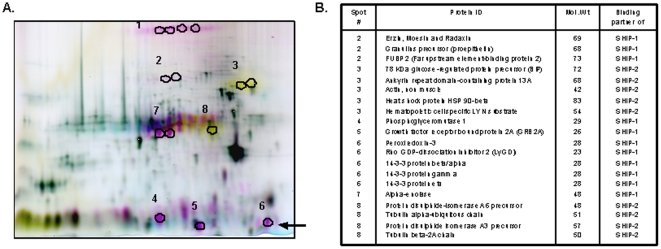
SHIP and SHIP-2 associate with unique binding partners. (A) DIGE was performed as described in Materials and Methods. Spots in green are control immunoprecipitations (IP), the spots in yellow are binding partners of SHIP, while spots in red are binding partners of SHIP-2. Black circles indicate the 8 spots that were cut out and subjected to in-gel trypsin digest followed by LC-MS/MS and protein identification using a database search. The black arrow shows the spot corresponding to LyGDI. (B) This table summarizes the binding partners of SHIP or SHIP-2 that were identified.

To identify non-overlapping binding partners of SHIP and SHIP-2 we used DIGE analysis. THP-1 cells were stimulated for 15 minutes with FcγRIIa/IIb-clustering antibodies and then lysed. SHIP and SHIP-2 immunoprecipitates were labeled, combined in equal ratios and separated using two dimensional SDS-PAGE (2D-PAGE). Based on fluorescence (SHIP-interacting proteins were red and SHIP-2 interacting proteins were yellow), eight unique spots were chosen for in-gel trypsin digest (Figure 1A) followed by LC-MS/MS mass spectrometry analysis. Twenty proteins were identified as potential binding partners of SHIP or SHIP-2 (Figure 1B) using a MASCOT Daemon database search. Two of these have been previously reported to associate with SHIP (spot #5, Grb2) [11] and SHIP-2 (spot #3 actin) [12] thus validating our methodology. Several proteins identified in this study such as 14-3-3, Ezrin, Radaxin and Moesin (ERM proteins), BiP (GRP78), HSP90β, protein disulphide isomerase LyGDI and tubulin [13]–[19] have been demonstrated to play an important role in actin dynamics during cytoskeleton remodeling, cellular adhesion and migration. The observation that SHIP and SHIP-2 associate with distinct signaling proteins involved in actin dynamics suggests that the two phosphatases may regulate different aspects of actin dynamics through non-catalytic mechanisms. Some proteins such as peroxiredoxin-3 and 14-3-3 can influence ROS production [20]–[22].

### LyGDI associates with SHIP

**Figure 2:**
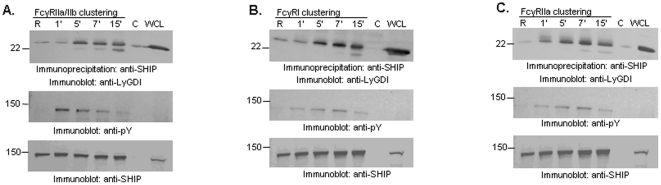
LyGDI is a novel binding partner of SHIP. (A) THP-1 cells were stimulated for the indicated time points using human FcγRIIa/IIb (CD32) antibody followed by goat F(ab')_2_ anti-mouse IgG. SHIP was immunoprecipitated from resting ‘R’ and activated ‘A’ cells and analyzed by Western blotting (IB) with anti-LyGDI (upper panel). Control IPs were performed using normal mouse IgG. The same membrane was reprobed with anti-phosphotyrosine (pY) (middle panel) and anti-SHIP (lower panel). Similar co-immunoprecipitation assays were performed from cells stimulated using (B) F(ab')_2_ fragments of FcγRI antibody 32.2 and goat F(ab')_2_ anti-mouse IgG secondary antibody, and (C) F(ab')_2_ fragments of FcγRIIa antibody IV.3 and goat F(ab')_2_ anti-mouse IgG secondary antibody.

Many proteins identified in this study, although important for regulating actin dynamics and ROS production, have not been previously reported downstream of FcγR activation. We verified several associations using co-immunoprecipitation (co-IP) assays (data not shown). For this study we chose to focus on LyGDI, a regulator of RhoGTPase activity that appeared as a binding partner of SHIP (spot #6). Because RhoGTPases play an important role in the actin cytoskeletal rearrangements associated with FcγR-mediated phagocytosis [23], [24], we explored the possibility that LyGDI regulates this process. First, to ascertain whether the SHIP-LyGDI association was physiologically relevant we performed co-IP assays. SHIP immunoprecipitates from FcγRIIa/IIb-stimulated THP-1 cells were analyzed by Western blotting for LyGDI. A time-dependent increase in the association between SHIP and LyGDI was observed (Figure 2A, upper panel). SHIP was also tyrosine phosphorylated under these condition as previously reported (Figure 2A, middle panel) [25], indicative of SHIP membrane translocation and activity. Equivalent loading of protein in all lanes was confirmed by reprobing the membranes with SHIP antibody (Figure 2A, lower panel).

Next, we assessed whether SHIP-LyGDI complex formation occurred downstream of other FcγR known to recruit and activate SHIP. We clustered FcγRI and FcγRIIa using receptor-specific F(ab')_2_ antibodies and measured SHIP-LyGDI association as described above. Results showed that SHIP inducibly associated with LyGDI downstream of both FcγRI and FcγRIIa, with similar kinetics (Figure 2B & C).

### FcγR clustering induces a SHIP-LyGDI multimolecular complex involving Shc and Grb2

**Figure 3:**
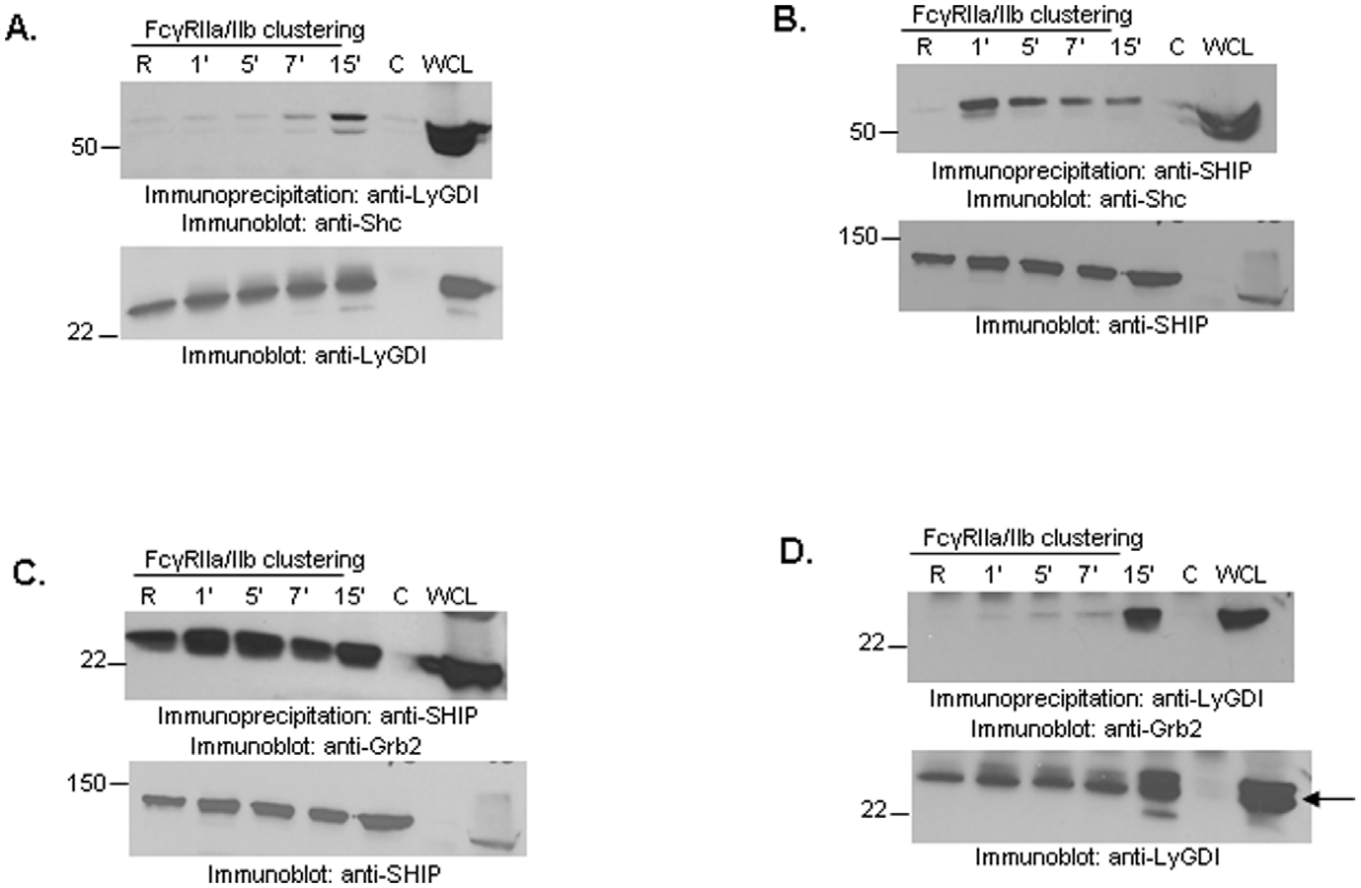
Shc and Grb2 are present in SHIP-LyGDI complexes upon FcγR clustering. THP-1 cells were stimulated for different time points using anti-CD32 followed by goat anti-mouse F(ab')_2_ IgG secondary antibody. (A) LyGDI was immunoprecipitated from resting and activated cells and analyzed by Western blotting with anti-Shc (upper panel). The same membrane was reprobed with anti-LyGDI to ensure equivalent protein loading (lower panel). Control IPs were done using normal goat IgG. (B) SHIP was immunoprecipitated from resting and activated cells and analyzed by Western blotting with anti-Shc (upper panel). The same membrane was reprobed with anti-SHIP to ensure equivalent protein loading (lower panel). Control IPs were done using normal mouse IgG. (C) SHIP was immunoprecipitated from resting and activated cells and analyzed by Western blotting with anti-Grb2 (upper panel). The same membrane was reprobed with anti-SHIP to ensure equivalent protein loading (lower panel). Control IPs were done using normal mouse IgG. (D) LyGDI was immunoprecipitated from resting and activated cells and analyzed by Western blotting with anti-Grb2 (upper panel). The same membrane was reprobed with anti-LyGDI to ensure equivalent protein loading (lower panel). Control IPs were done using normal goat IgG. All co-IP results are representative of at least three independent experiments.

We then examined downstream molecules of FcγR that may be responsible for this SHIP-LyGDI complex formation. The first candidate was Shc, as T-cell receptor activation is known to induce LyGDI-Shc association in Jurkat cells [26]. In accordance with this we found that FcγR clustering in THP-1 cells led to LyGDI-Shc association (Figure 3A). FcγR clustering has been shown to induce association between SHIP and Shc (Figure 3B), while Grb2 is constitutively associated with SHIP (Figure 3C) [27], [28]. Consistent with this, we found that Grb2 co-precipitated with LyGDI upon FcγR clustering (Figure 3D).

### Grb2 is necessary for FcγR-mediated SHIP-LyGDI association

**Figure 4:**
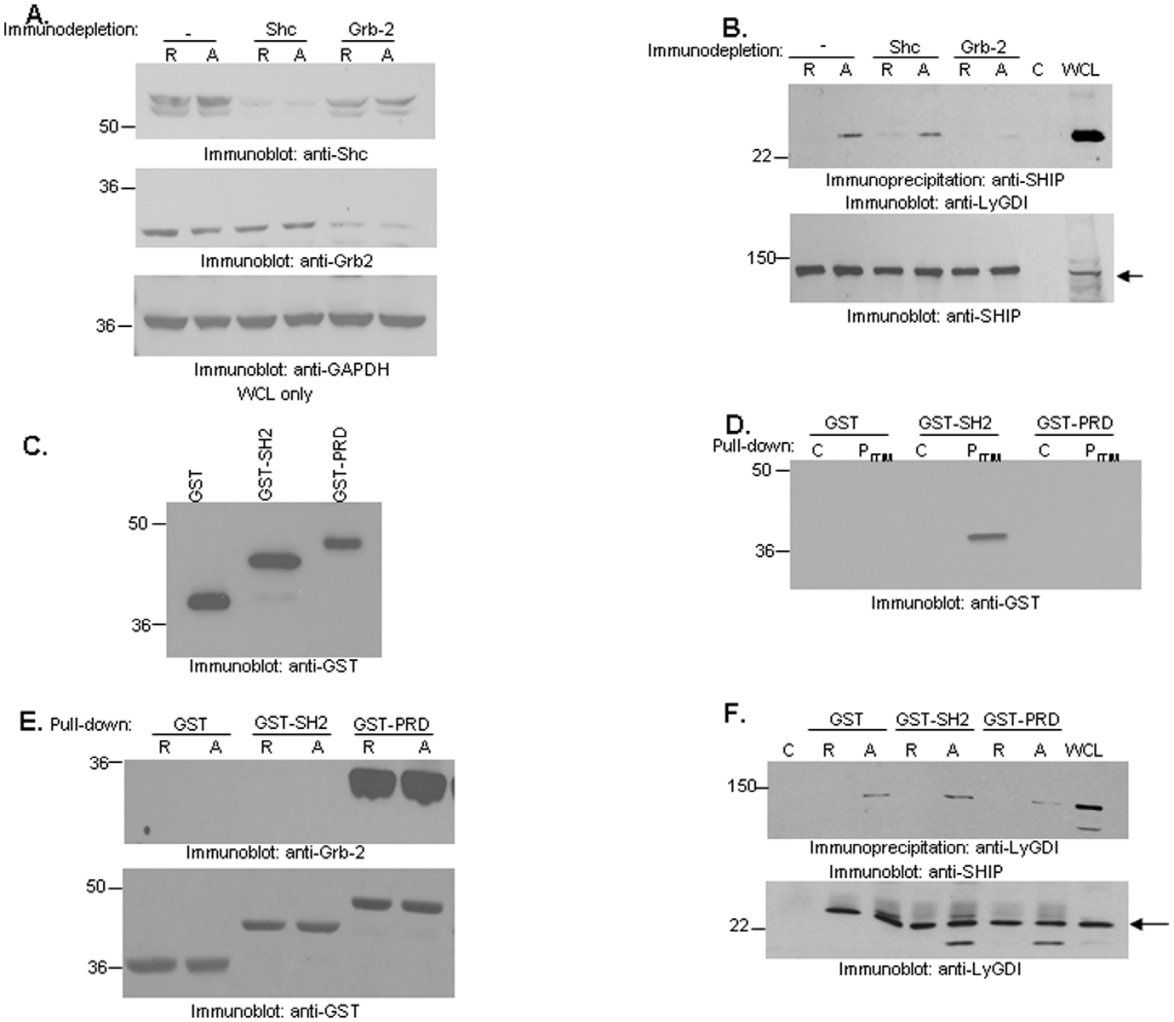
Grb2 is necessary for FcγR-induced SHIP-LyGDI association. THP-1 cells were stimulated for 15 minutes using CD32 antibody followed by goat anti-mouse IgG secondary antibody. Lysates were made and immunodepleted for Shc or Grb2. (A) Non-depleted, Shc-depleted and Grb2-depleted lysates from resting and activated cells were analyzed by Western blotting with anti-Shc (upper panel) and anti-Grb2 (middle panel). The same membrane was reprobed with anti-GAPDH (lower panel) to ensure equivalent protein loading. (B) SHIP was immunoprecipitated from resting and activated immunodepleted lysates and analyzed by Western blotting with LyGDI antibody (upper panel). The same membrane was reprobed with anti-SHIP to ensure equivalent protein loading (lower panel). Control IPs were done using normal mouse IgG. (C) 0.5 µg of purified GST, SHIP-GST-SH2 and SHIP-GST-PRD fusion proteins were analyzed by Western blotting with anti-GST antibody. (D) N-terminal biotinylated control peptide ‘C’ or phosphorylated peptide containing to the FcγRIIb ITIM sequence (P_ITIM_) immobilized on NeutrAvidin beads were used to pulldown SHIP-GST-SH2, SHIP-GST-PRD or GST alone from solution. Bead-bound proteins were analyzed by Western blotting with GST antibody. (E) GST, SHIP-GST-SH2 or SHIP-GST-PRD were added to resting ‘R’ or FcγRIIa/IIb-stimulated ‘A’ THP-1 lysates and GST pulldowns were performed by adding glutathione agarose beads. Bead-bound proteins were analyzed by Western blotting with anti-Grb2 (upper panel). The same membrane was reprobed with anti-GST (lower panel). (F) GST, SHIP-GST-SH2 or SHIP-GST-PRD were added to resting ‘R’ or FcγRIIa/IIb-stimulated ‘A’ THP-1 lysates and LyGDI was immunoprecipiated. The immunoprecipitates were analyzed by Western blotting for SHIP (upper panel). The same membrane was reprobed with anti-LyGDI antibody (lower panel).

To further decipher the role of Shc and Grb2 in mediating the SHIP-LyGDI association, we performed immunodepletion studies. Shc (Figure 4A, upper panel) and Grb2 (Figure 4A, middle panel) were immunodepleted from resting and FcγRIIa/IIb-stimulated THP-1 lysates. We then immunoprecipitated SHIP from the immunodepleted lysates and probed for LyGDI by Western blotting. Results showed that FcγR-induced SHIP-LyGDI association occurred in non-depleted lysates (lane 2). Surprisingly, we also saw this association in Shc-depleted lysates upon FcγR activation. However, in Grb2-depleted lysates (Figure 4B, upper panel, lane 6) SHIP-LyGDI association was dramatically reduced. This suggests that, although Shc and Grb2 are both present in the SHIP-LyGDI complex, only Grb2 is required for LyGDI association.

Grb2 contains a central SH2 domain that is flanked by an SH3 (Src Homology 3) domain on either side. SHIP interaction with Grb2 is mediated via the PRD of SHIP. Because Grb2 is necessary for SHIP-LyGDI association, we hypothesized that FcγR clustering led to an indirect association between SHIP and LyGDI via Grb2, which depended on the C-terminal PRD of SHIP.

To test this hypothesis we generated GST-SH2 and GST-PRD fusion constructs of SHIP using standard subcloning techniques followed by overexpression and purification from *E.coli* BL21(DE3) (Figure 4C). Next, we determined whether SHIP GST-SH2 and SHIP GST-PRD fusion proteins were functional. In our peptide pulldown assays using non-phosphorylated peptide (C) or peptide containing phosphorylated ITIM of FcγRIIb (P_ITIM_), we found that the SHIP GST-SH2 domain specifically associated with P_ITIM_ but not SHIP GST-PRD or GST alone (Figure 4D). This was as previously reported [29]. Next, we observed that Grb2 associated with SHIP GST-PRD in both resting and FcγRIIa/IIb-stimulated THP-1 lysates. However, it did not associate with SHIP GST-SH2 (Figure 4E, upper panel), in accordance with previous studies [30]. Anti-GST antibody reprobes of the same membrane confirmed that equivalent amounts of GST proteins were used (Figure 4E, lower panel).

Next, to access whether SHIP-LyGDI association required the C-terminal PRD of SHIP, we performed competition assays where an excess of SHIP GST-SH2 or SHIP GST-PRD was added to resting and FcγRIIa/IIb-stimulated THP-1 lysates. LyGDI immunoprecipitates from these lysates were probed for SHIP by Western blotting. SHIP-LyGDI association could be detected in the presence of SHIP GST-SH2 or GST alone but was dramatically reduced in the presence of SHIP GST-PRD (Figure 4F, upper panel). Membranes were reprobed with anti-LyGDI as a loading control (Figure 4F, lower panel). Data from three independent experiments were analyzed by Student's t-test. No significant difference in SHIP-LyGDI association was observed in presence of SHIP GST-SH2 or GST alone. However, SHIP GST-PRD was able to disrupt SHIP-LyGDI interaction (p-value, 0.053). These results suggest that the C-terminal PRD of SHIP is required for mediating the SHIP-LyGDI association.

### LyGDI influences Rac localization downstream of FcγR activation

**Figure 5:**
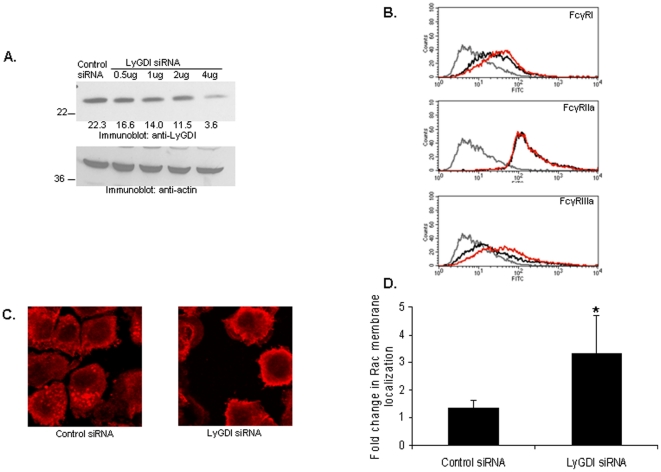
LyGDI influences Rac membrane localizaation. (A) 1×10^7^ PBM were transfected with either scrambled or LyGDI siRNA. Western blotting was done to measure LyGDI after 48 hours (upper panel). The membrane was reprobed with anti-actin to ensure equivalent loading (lower panel). Numbers in the upper panel are mean optical density (arbitrary units) normalized to actin. (B) Control and LyGDI siRNA-transfected samples were tested for FcγR expression by flow cytometery analysis. Overlay histograms show FcγR staining in secondary-only antibody controls (gray), control siRNA-transfected (black) and si-LyGDI-transfected (red). (C) Control and LyGDI siRNA-transfected PBMs were stained with mouse anti-Rac followed by Alexa flour 555 conjugated to goat anti-mouse IgG, F(ab')_2_ fragment. (D) The fold change in Rac membrane localization in control versus LyGDI siRNA-transfected PBMs was quantified as described in Materials and Methods.

LyGDI is a guanidine dissociation inhibitor for RhoGTPases such as Rac. Previous *in vitro* studies using recombinant LyGDI demonstrated that LyGDI can negatively regulate the biochemical activity of RhoGTPases [9]. Hence, we next examined the influence of LyGDI on Rac localization in human monocytes. We knocked down LyGDI in PBM using nucleofection (Figure 5A) and verified cell viability using trypan blue staining. To rule out the possibility that knockdown affected expression of FcγR, we stained for FcγR and analyzed receptor levels using flow cytometry (Figure 5B).

We fluorescently labeled Rac and examined its localization in control and knockdown cells. We found distinct punctate areas in the cytosol of control siRNA-transfected PBM. However upon LyGDI knockdown there was a reduction in these areas with a concurrent increase in membrane localization (Figure 5C & 5D). Because membrane association of Rac follows Rac activation and is required for downstream activation of Rac effectors [8], [31], [32], these results suggest that LyGDI negatively regulates Rac activation by sequestering Rac within the cytosol.

### LyGDI negatively regulates FcγR-mediated phagocytosis

**Figure 6:**
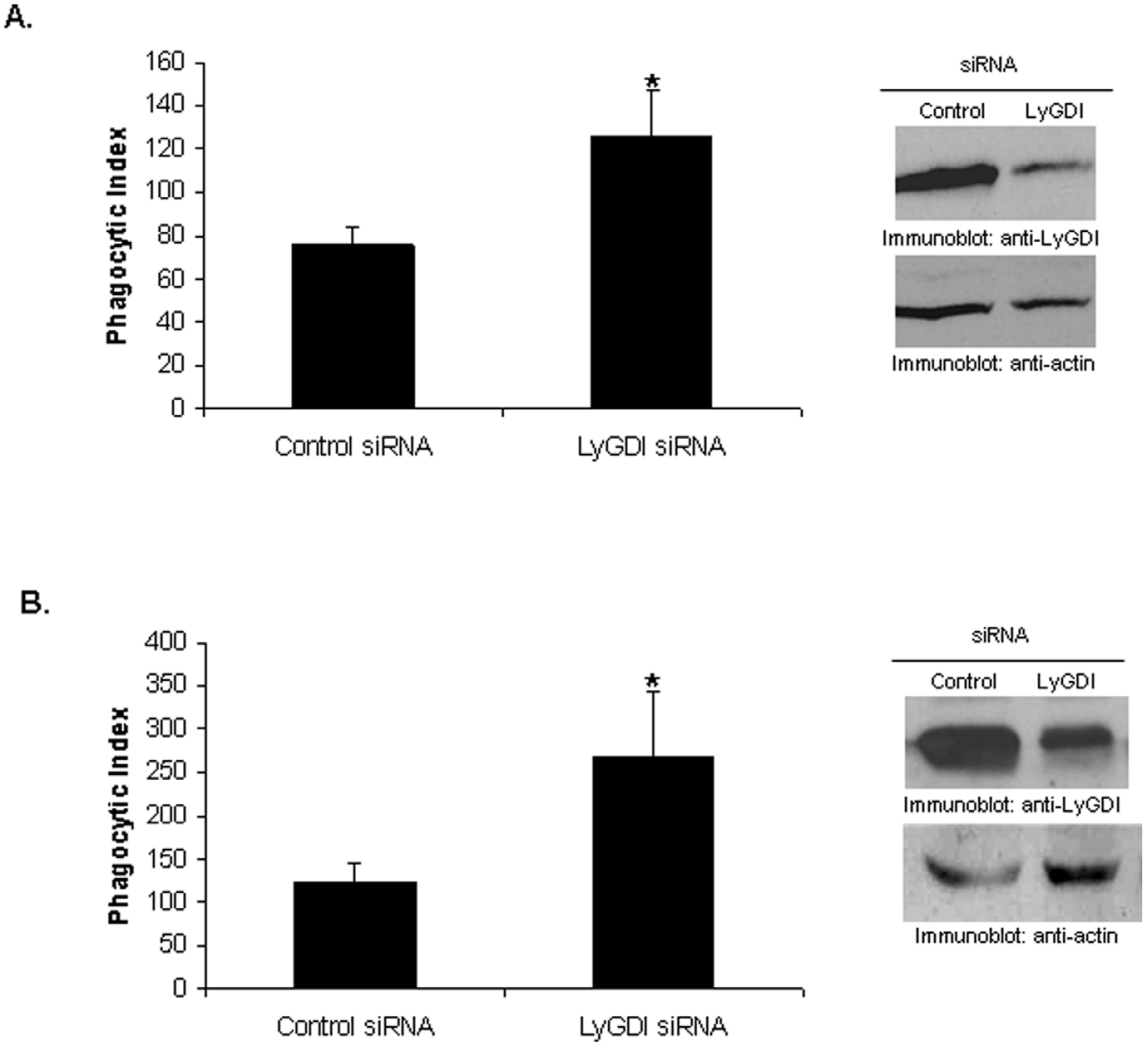
LyGDI is a negative regulator of FcγR-mediated phagocytosis. Control and LyGDI siRNA-transfected THP-1 (A) or PBM (B) were incubated with fluoresceinated, IgG-coated sheep red blood cells (SRBC) at 37°C for 40 minutes. Phagocytosis was measured by counting the total number of SRBC ingested by 100 transfectants and is plotted as the phagocytic index. Three independent experiments were performed and scored in a blinded fashion. Data were analyzed by Student's t-test (* denotes p<0.05).

Rac activation is necessary for promoting actin assembly and subsequent phagocytosis downstream of FcγR activation [23], [24]. Hence, we predicted that LyGDI would have an effect on FcγR-mediated phagocytosis. We transfected THP-1 with either control or LyGDI siRNA and measured phagocytosis of antibody-coated sheep red blood cells (SRBC). We found a significant increase in the phagocytic index of cells knocked down for LyGDI (Figure 6A).

Next, we confirmed these findings in primary human monocytes. PBM from 6 donors were subjected to the same siRNA and phagocytosis assays. As shown in Figure 6B, knockdown of LyGDI led to a significant increase in the phagocytic index. These results strongly suggest that LyGDI is a negative regulator of FcγR-mediated phagocytosis.

## Discussion

SHIP and SHIP-2 are important regulatory phosphatases that play distinct roles despite similar catalytic activity, as suggested by *in vivo* studies [33], [34]. They are very similar in domain organization, overall structural features and catalytic properties [27], [35], [36]. Each contains an N-terminal SH2 domain, the central catalytic domain that catalyzes hydrolysis of PtdIns(3,4,5)P_3_, a key secondary messenger in the PI3K pathway, and a C-terminal PRD harboring NPXY motifs. Despite their high degree of sequence homology within the catalytic regions and similar enzymatic activity, SHIP and SHIP-2 have largely divergent non-catalytic domains that result in differential association with signaling proteins. This suggests that they may serve both overlapping and non-overlapping functions. For example, previous studies have demonstrated that SHIP associates with Grb2, a protein involved in growth factor signaling, whereas SHIP-2 associates with Abl [11]. Likewise, it was demonstrated that only SHIP-2 associates with actin-binding proteins such as filamin and p130Cas that are involved in cytoskeletal rearrangement [37], [38]. Associations mediated by the non-catalytic domains are known to influence key signaling pathways in B cells and macrophages. In macrophages, SHIP downregulation of FcγR-mediated IL-1β production is independent of catalytic activity but requires functional non-catalytic domains [39]. In B cells SHIP exerts a negative influence on the Ras signaling pathway by binding to phosphorylated Shc via its non-catalytic domain, thereby sequestering binding sites for the Grb2/Sos complex [28], [40]. Additionally, SHIP's association with p62Dok via its non-catalytic domain results in hyper-phosphorylation of p62Dok, turning off Ras signaling through the activation of RasGAP [41]. Therefore, protein interactions mediated by the non-catalytic domains of SHIP(s) have important biological consequences.

To identify as-yet undiscovered proteins that uniquely associate with SHIP or SHIP-2 we have made use of DIGE analysis. DIGE is traditionally used to quantify relative abundances and monitor differences in expression profiles of proteins in a given sample [42]. Here, we have combined the selectivity of co-IPs with the sensitivity of DIGE to directly compare the association profiles of two closely related protein SHIP and SHIP-2. The system is not quantitative because of the nature of co-IP but we were able to identify several proteins that uniquely associated with either SHIP or SHIP-2 upon FcγR activation. Not surprisingly, many have been reported to play an important role in mediating actin dynamics. For example, ERM proteins have been shown to be important for polymerization and organization of actin filaments [13]–[15]. They also inhibit stress fibers by blocking RhoA. BiP (GRP78) promotes cell spreading and cell polarity by influencing Focal Adhesion Kinase (FAK) activity [16]. Protein disulphide isomerases are important for cell migration and invasion [17].14-3-3 is an adaptor molecule that can influence actin dynamics by associating with other proteins. Association of 14-3-3 with IRSp53 can modulate filopodial dynamics while it influences actin stress fiber formation through association with ankyrin repeat containing protein [18]. 14-3-3 can also disrupt cell migration upon association with MK5 [19].

Both SHIP and SHIP-2 are recruited to phagocytic cups upon FcγR clustering and are known to influence actin dynamics indirectly through SHIP's catalytic activity [2], [43]–[45]. This collectively suggests that SHIP proteins may utilize both catalytic as well as non-catalytic mechanisms for controlling actin dynamics during cellular activation. The observation that SHIP and SHIP-2 associate with distinct signaling intermediates suggests that the two proteins may regulate different aspects of actin dynamics. Understanding such differences will not only enhance our understanding of FcγR-mediated signaling responses but also allow us to design more specific therapeutic modulators to selectively alter SHIP functions without altering the overall catalytic function within the context of a pathological condition.

Some of the other proteins that were identified as binding partners of SHIP(s) such as progranulin [20], peroxoredoxin-3 [21] and 14-3-3 [22] have a role in regulation of reactive oxygen species (ROS) generation. ROS production accompanies FcγR-mediated phagocytosis and is tightly regulated by SHIP catalytic activity. Therefore, association of SHIP(s) with different signaling molecules can alter various functions downstream of FcγR activation. However, the effects of these differential associations remain to be tested.

Our results suggest that the FcγR-mediated association of LyGDI with SHIP required Grb2. The hydrolysis of PtdIns(3,4,5)P_3_ by SHIP is known to downregulate the activity of proteins such as Vav, thereby decreasing Rac activation and subsequent phagocytosis [46]. We speculate that association between SHIP and LyGDI points to a novel, non-catalytic means by which SHIP may additionally downregulate FcγR activity by stabilizing LyGDI-Rac association. Further studies need to be done to test this hypothesis.

FcγR function has been implicated in both the pathophysiology of autoimmune diseases and in mediating cytotoxic effects of monoclonal anti-tumor antibodies [47]–[49]. However, targeting its downstream regulator SHIP has proven difficult. Results from this study have uncovered another molecule that plays a role in SHIP-mediated regulation and that might be a putative target for therapy.

## Materials and Methods

### Cell antibodies and reagents

THP-1 cells were obtained from the ATCC and maintained in RPMI supplemented with 5% fetal bovine serum. Anti-Rac antibody was purchased from Chemicon International (Temecula, CA). Phospho-tyrosine (pY) and SHIP antibodies were from Cell Signaling Technology (Beverly, MA). Human CD32 (FcγRIIa/IIb) antibody was purchased from BD Pharmingen (San Diego, CA). The F(ab')_2_ of human FcγRI (32.2), human FcγRIIa (IV.3) and human FcγRIIIa (3 g8) antibody were obtained from Medarex (Princeton, NJ). Goat anti-mouse F(ab')_2_ antibody was from Jackson ImmunoResearch (West Grove, PA). LyGDI, actin and HRP-conjugated secondary antibodies were from Santa Cruz Biotechnology (Santa Cruz, CA). siRNA constructs for LyGDI were also from Santa Cruz.

### Immunoprecipitation, Immunodepletion and Western blotting

THP-1 cells were activated using monoclonal F(ab')_2_ antibodies against FcγRI (32.2), FcγRIIa (IV.3) or FcγRIIa/IIb (CD32) followed by goat F(ab')_2_ anti-mouse IgG secondary antibody. Resting and activated cells were lysed in TN1 lysis buffer and post-nuclear lysates were incubated overnight with the specified antibodie followed by 60-minute incubation at 4°C with protein G-agarose beads (Gibco BRL, Grand Island, NY). Immunoprecipitations with appropriate control antibodies were performed in lysates from resting cells. After incubation, beads were washed in TN1 lysis buffer, boiled in SDS sample buffer for 5 minutes and subjected to SDS/PAGE. Proteins were transferred to nitrocellulose filter probed with the specified antibodies and developed by enhanced chemiluminescence. For immunodepletion studie the protein of interest was immunodepleted from resting and activated THP-1 lysates by incubating overnight with the specified antibodies and protein-G-agarose beads.

### Generation of GST-SHIP SH2 and PRD DNA contructs

DNA sequences for the SHIP SH2 and PRD domains were isolated by polymerase chain reaction (PCR) from pcDNA3.1-SHIP plasmid (kindly provided by Dr. K. Mark Coggeshall). Amplification and addition of EcoR1 and Xho1 restriction sites was done using the following primers: N-SH2, 5′-GGAATTCATGGCCTCAGTGTGTGGGACAC-3′ and 5′-ACCGGCTCGAGGTCTGGCTCTCTCTCCCCCTC-3′; C-PRD, 5′-GGAATTCTCCAGTGGAATGAAATGCTTGAAG-3′ and 5′-ACCGGCTCGAGTCACTGCATGGCAGTCCTGCC-3′. PCR products were digested with *Eco*RI and Xho1, then ligated into pGEX-4T-1(Amersham bioscience Piscataway, NJ) to generate GST fusion constructs. All final constructs were analyzed by dideoxy sequencing.

### Purification of GST-SH2 and GST-PRD SHIP recombinant proteins

*E. coli* BL21(DE3) cells were transformed with either pGEX-4T-1-SH2 SHIP or pGEX-4T-1-PRD SHIP plasmids and grown in LB medium to the mid-log phase, then induced by 300 µM isopropyl β-d-thiogalactoside (IPTG) for 3 h at 30°C. Cells were harvested by centrifugation, resuspended in lysis buffer (10 mM Na_2_HPO_4_, pH 7.2, 1 mM EDTA, 1 mg/mL aprotonin and leupeptin, 1 mg/mL of p122 protease cocktail inhibitor, 1 mg/mLlysozyme and 5 mg/mL of protamine sulphate) followed by sonication on ice. GST fusion proteins were purified from crude lysates on pre-equlibriated GST agarose columns as previously described [50]. Eluted proteins were dialyzed overnight in dialysis buffer (50 mM Tri pH 8.0, 100 mM NaCl and 1 mM EDTA) and concentrated in an Amicon concentrator to approximately 3.5 mg/mL. After concentration and addition of glycerol (final 33%), protein was snap-frozen in liquid nitrogen and stored at −80°C.

### GST pulldown Assays

Lysates from resting and FcγRIIa/IIb-stimulated THP-1 cells were incubated with 5 µg of GST, SHIP-GST-SH2 or SHIP-GST-PRD plus a 50 µl slurry of glutathione-agarose beads (Sigma, Saint Loui MO). Samples were rocked overnight at 4°C. Beads were washed twice with TN1 lysis buffer and analyzed by Western blotting using the specified antibodies.

### Peptide pulldown Assays

N-terminal biotinylated control peptide, C- (ETADGGYMTLNPRAPTDDDKNIYLTLG) and N-terminal biotinylated peptide containing the phophorylated ITIM sequence of FcγRIIb, P_ITIM_ (EAENTITpYSLLKH) with 95% purity were from Invitrogen (Carlsbad, CA). 1.0 µg of GST, SHIP-GST-SH2 or SHIP-GST-PRD was added to 1 mL of TN1 lysis buffer containing 5 µg of N-terminal biotinylated control peptide (non-phosphorylated) or the phospho-peptide and rocked at 4°C for 2 hours. Subsequently, the peptides were pulled down by addition of 25 µL of NeutrAvidin beads (Thermo Fisher Scientific Inc., Rockford, IL) and incubated on a rocker at 4°C for an additional hour. Beads were collected, washed twice in TN1 lysis buffer and analyzed by Western blotting using the specified antibodies.

### Competition assays

THP-1 cells were either left unstimulated or stimulated for 15 minutes with FcγRIIa/IIb (CD32) plus goat anti-mouse F(ab')_2_ antibody. Protein lysates were made and incubated on a rocker for 2 hours at 4°C with 5 µg of GST, SHIP-GST-SH2 or SHIP-GST-PRD or GST plus 2 µg of anti-LyGDI. 30 µL of protein G-agarose beads were then added and the lysates incubated for an additional hour. Beads were then collected, washed twice with TN1 lysis buffer and boiled in SDS sample buffer for analysis by Western blotting. Western blots were quantitated using ImageJ software (NIH).

### Isolation of PBM

CD14-positive PBM were isolated from buffy coats using the MACS monocyte isolation kit (Miltenyi Biotech, Auburn, CA) as described previously [51]. The purity of monocytes was >97% as assessed by flow cytometry using anti-CD14 staining.

### Transfections

THP-1 or PBM were transfected with siRNA constructs using the Amaxa Nucleofector apparatus (Amaxa biosystem Colonge, Germany). Briefly, 1×10^7^ cells were resuspended in 100 µL of Cell Line Nucleofector Solution T (Amaxa Biosystems) and nucleofected with either scrambled or LyGDI siRNA. Immediately after transfection the cells were transferred to 10 mL of pre-warmed media (RPMI supplemented with 10% FBS) and subsequently cultured in 12-well plates for 48 hours at 37°C. For PBM the media was supplemented with M-CSF (20 ng/mL).

### Flow cytometry analysis for FcγR expression

Control and LyGDI siRNA-transfected samples were tested for FcγR expression by incubating with F(ab')_2_ antibodies against FcγRI (32.2), FcγRIIa (IV.3) or FcγRIIIa (3 g8) at the concentration of 1 µg/mL for 30 minutes at 4°C. Cells were washed twice with PBS and then incubated with FITC-conjugated goat F(ab')_2_ anti-mouse IgG secondary antibody for 30 minutes at 4°C. After washing again with PBS they were fixed in 1% Paraformaldehyde (PFA) and analyzed by flow cytometry on a FACS Calibur flow cytometer (BD Bioscience San Jose, CA), recording 10,000 events per condition.

### Fluorescence Microscopy

Control and LyGDI siRNA-transfected PBMs were fixed in 4% PFA and permeabilized with 0.2% triton X-100. After blocking with goat serum the cells were stained with mouse anti-Rac antibody followed by Alexa flour 555 conjugated to goat anti-mouse IgG, F(ab')2 fragment to detect Rac. Samples were read using a Zeiss Meta 510 multiphoton confocal microscope (Carl Zeis Jena, Germany) for enhanced resolution. Quantification of Rac protein was achieved using Adobe Photoshop CS5 Histogram analysis as previously described [52]. Briefly, the maximal (Rac: Alexa flour 555-red) level was set on each image to standardize protein expression per image using the information setting for RGB. After, a macro was set-up to determine unbiased protein expression in either the cytosol or at the cell periphery. The designated red pixels are converted to black using: Color Select - Color Range - Inverse - Delete - Threshold - Histogram, and the percent of black pixels per field is recorded. Using the batch feature, all images are quantified in the same manner by the software.

### Phagocytosis Assays

Phagocytosis assays were performed as previously described [51]. Briefly, IgG-coated PKH26-labeled sheep red blood cells (SRBC) were added to siRNA-transfected THP-1 or PBM. Cells were pelleted by low-speed centrifugation followed by incubation for 40 minutes at 37°C. Cells were then subjected to brief hypotonic lysis with water prior to fixation in PFA and analyzed by fluorescence microscopy in a blinded fashion. Phagocytic index was defined as the total number of RBCs ingested by 100 phagocytes.

### DIGE (Differential in gel electrophoresis)

#### A) Cydye labeling

SHIP and SHIP-2 were immunoprecipitated (IP) from FcγRIIa/IIb stimulated THP-1 cells for 15 minutes as described above. Proteins were extracted from the immunoprecipitates using extraction buffer (8 M urea and 4% CHAPS), precipitated with trichloroacetic acid and resuspended in Tris buffer (30 mM Tris pH 8.5, 8 M urea and 4% CHAPS) for cyanine dye labeling. Under similar condition control IP for SHIP and SHIP-2 were performed using normal mouse and normal goat IgG respectively. Monofunctional CyDyes NHS esters (GE Healthcare, Pataskala, OH) were used with three different fluorescent colors (Cy3, Cy5 and Cy2) for SHIP IP, SHIP-2 IP and control IP respectively, incubated for 30 minutes at room temperature and the reaction was quenched by addition of 1 µL of 10 mM lysine. **B) Isoelectric focusing (IEF):** The CyDye-labeled samples were mixed in a 1∶1∶1 ratio, and used to rehydrate immobilon pH 3–10 strips overnight under mineral oil. The strips were focused on an IPGphorII IEF under mineral oil in a ceramic manifold. After focusing, the strips were equilibrated in equilibration buffer A (50 mM Tris-Cl pH 8.8, 6 M Urea, 30% glycerol, 1.0% saturated bromophenol blue solution, 2% SD 65 mM DTT) for 15 minute followed by equilibration buffer B (50 mM Tris-Cl pH 8.8, 6 M Urea, 30% glycerol, 1.0% saturated bromophenol blue solution, 2% SD 135 mM iodoacetamide) for 15 minutes and rinsed in 1x SDS-loading buffer. The strips were then placed in an 8% SDS-PAGE minigel sealed in place with sealing solution (0.5% agarose, 1.0% saturated bromophenol blue solution, 98.5% SDS-PAGE running buffer) and then subjected to SDS-PAGE. Gels were rinsed with water and immediately scanned for the three CyDye wavelengths. Eight spots were cut out and subjected to in-gel digest with trypsin followed by LC-MS/MS (Thermo Finnigan LTQ, Thermo Scientific Inc., Odessa, TX) and protein identification using database search (MASCOT Daemon, Matrix science, Boston, MA).

#### Statistical Analysis

Data were analyzed using Student's t*-* test to test for statistically significant differences, where appropriate.

## References

[1] Aderem A, Underhill DM (1999). Mechanisms of phagocytosis in macrophages.. Annu Rev Immunol.

[2] Cox D, Dale BM, Kashiwada M, Helgason CD, Greenberg S (2001). A regulatory role for Src homology 2 domain-containing inositol 5′-phosphatase (SHIP) in phagocytosis mediated by Fc gamma receptors and complement receptor 3 (alpha(M)beta(2); CD11b/CD18).. J Exp Med.

[3] Pengal RA, Ganesan LP, Fang H, Marsh CB, Anderson CL (2003). SHIP-2 inositol phosphatase is inducibly expressed in human monocytes and serves to regulate Fcgamma receptor-mediated signaling.. J Biol Chem.

[4] Hejna JA, Saito H, Merkens L Tittle TV, Jakobs PM (1995). Cloning and characterization of a human cDNA (INPPL1) sharing homology with inositol polyphosphate phosphatases.. Genomics.

[5] Erneux C, Govaerts C, Communi D, Pesesse X (1998). The diversity and possible functions of the inositol polyphosphate 5-phosphatases.. Biochim Biophys Acta.

[6] Lelias JM, Adra CN, Wulf GM, Guillemot JC, Khagad M (1993). cDNA cloning of a human mRNA preferentially expressed in hematopoietic cells and with homology to a GDP-dissociation inhibitor for the rho GTP-binding proteins.. Proc Natl Acad Sci U S A.

[7] Dovas A, Couchman JR (2005). RhoGDI: multiple functions in the regulation of Rho family GTPase activities.. Biochem J.

[8] Bishop AL, Hall A (2000). Rho GTPases and their effector proteins.. Biochem J.

[9] Scherle P, Behrens T, Staudt LM (1993). Ly-GDI, a GDP-dissociation inhibitor of the RhoA GTP-binding protein, is expressed preferentially in lymphocytes.. Proc Natl Acad Sci U S A.

[10] Leffers H, Nielsen M, Andersen AH, Honore B, Madsen P (1993). Identification of two human Rho GDP dissociation inhibitor proteins whose overexpression leads to disruption of the actin cytoskeleton.. Exp Cell Res.

[11] Wisniewski D, Strife A, Swendeman, Erdjument-Bromage H, Geromanos S (1999). A novel SH2-containing phosphatidylinositol 3,4,5-trisphosphate 5-phosphatase (SHIP2) is constitutively tyrosine phosphorylated and associated with src homologous and collagen gene (SHC) in chronic myelogenous leukemia progenitor cells.. Blood.

[12] Dyson JM, O′Malley CJ, Becanovic J, Munday AD, Berndt MC (2001). The SH2-containing inositol polyphosphate 5-phosphatase, SHIP-2, binds filamin and regulates submembraneous actin.. J Cell Biol.

[13] Faure Salazar-Fontana LI, Semichon M, Tybulewicz VL, Bismuth G (2004). ERM proteins regulate cytoskeleton relaxation promoting T cell-APC conjugation.. Nat Immunol.

[14] Lee JH, Katakai T, Hara T, Gonda H, Sugai M (2004). Roles of p-ERM and Rho-ROCK signaling in lymphocyte polarity and uropod formation.. J Cell Biol.

[15] Li Y, Harada T, Juang YT, Kyttaris VC, Wang Y (2007). Phosphorylated ERM is responsible for increased T cell polarization, adhesion, and migration in patients with systemic lupus erythematosus.. J Immunol.

[16] Su R, Li Z, Li H, Song H, Bao C (2010). Grp78 promotes the invasion of hepatocellular carcinoma.. BMC cancer.

[17] Goplen D, Wang J, Enger PO, Tysnes BB, Terzis AJ (2006). Protein disulfide isomerase expression is related to the invasive properties of malignant glioma.. Cancer Res.

[18] Robens JM, Yeow-Fong L, Ng E, Hall C, Manser E (2010). Regulation of IRSp53-dependent filopodial dynamics by antagonism between 14-3-3 binding and SH3-mediated localization.. Mol Cell Biol.

[19] Tak H, Jang E, Kim SB, Park J, Suk J (2007). 14-3-3epsilon inhibits MK5-mediated cell migration by disrupting F-actin polymerization.. Cell Signal.

[20] Zhu J, Nathan C, Jin W, Sim D, Ashcroft GS (2002). Conversion of proepithelin to epithelins: roles of SLPI and elastase in host defense and wound repair.. Cell.

[21] Bokoch GM, Diebold B, Kim J, Gianni D (2009). Emerging evidence for the importance of phosphorylation in the regulation of NADPH oxidases.. Antioxid Redox Signal.

[22] Chen L, Na R, Gu M, Salmon AB, Liu Y (2008). Reduction of mitochondrial H2O2 by overexpressing peroxiredoxin 3 improves glucose tolerance in mice.. Aging Cell.

[23] Hoppe AD, Swanson JA (2004). Cdc42, Rac1, and Rac2 display distinct patterns of activation during phagocytosis.. Mol Biol Cell.

[24] Yamauchi A, Kim C, Li Marchal CC, Towe J (2004). Rac2-deficient murine macrophages have selective defects in superoxide production and phagocytosis of opsonized particles.. J Immunol.

[25] Tu Z, Ninos JM, Ma Z, Wang JW, Lemos MP (2001). Embryonic and hematopoietic stem cells express a novel SH2-containing inositol 5′-phosphatase isoform that partners with the Grb2 adapter protein.. Blood.

[26] Groysman M, Hornstein I, Alcover A, Katzav S (2002). Vav1 and Ly-GDI two regulators of Rho GTPase function cooperatively as signal transducers in T cell antigen receptor-induced pathways.. J Biol Chem.

[27] Kavanaugh WM, Pot DA, Chin SM, Deuter-Reinhard M, Jefferson AB (1996). Multiple forms of an inositol polyphosphate 5-phosphatase form signaling complexes with Shc and Grb2.. Curr Biol.

[28] Tridandapani S, Pradhan M, LaDine JR, Garber Anderson CL (1999). Protein interactions of Src homology 2 (SH2) domain-containing inositol phosphatase (SHIP): association with Shc displaces SHIP from FcgammaRIIb in B cells.. J Immunol.

[29] Muraille E, Bruhns P, Pesesse X, Daeron M, Erneux C (2000). The SH2 domain containing inositol 5-phosphatase SHIP2 associates to the immunoreceptor tyrosine-based inhibition motif of Fc gammaRIIB in B cells under negative signaling.. Immunol Lett.

[30] Tridandapani S, Phee H, Shivakumar L, Kelley TW, Coggeshall KM (1998). Role of SHIP in FcgammaRIIb-mediated inhibition of Ras activation in B cells.. Mol Immunol.

[31] del Pozo MA, Alderson NB, Kiosses WB, Chiang HH, Anderson RG (2004). Integrins regulate Rac targeting by internalization of membrane domains.. Science.

[32] del Pozo MA, Price L, Alderson NB, Ren XD, Schwartz MA (2000). Adhesion to the extracellular matrix regulates the coupling of the small GTPase Rac to its effector PAK.. EMBO J.

[33] Damen JE, Ware MD, Kalesnikoff J, Hughes MR, Krystal G (2001). SHIP's C-terminus is essential for its hydrolysis of PIP3 and inhibition of mast cell degranulation.. Blood.

[34] Sleeman MW, Wortley KE, Lai KM, Gowen LC, Kintner J (2005). Absence of the lipid phosphatase SHIP2 confers resistance to dietary obesity.. Nat Med.

[35] Lioubin MN, Algate PA, Tsai Carlberg K, Aebersold A (1996). p150Ship, a signal transduction molecule with inositol polyphosphate-5-phosphatase activity.. Genes Dev.

[36] Prasad N, Topping R, Decker SJ (2002). Src family tyrosine kinases regulate adhesion-dependent tyrosine phosphorylation of 5′-inositol phosphatase SHIP2 during cell attachment and spreading on collagen I.. J Cell Sci.

[37] Prasad N, Topping R, Decker SJ (2001). SH2-containing inositol 5′-phosphatase SHIP2 associates with the p130(Cas) adapter protein and regulates cellular adhesion and spreading.. Mol Cell Biol.

[38] Ganesan LP, Joshi T, Fang H, Kutala VK, Roda J (2006). FcgammaR-induced production of superoxide and inflammatory cytokines is differentially regulated by SHIP through its influence on PI3K and/or Ras/Erk pathways.. Blood.

[39] Dunant NM, Wisniewski D, Strife A, Clarkson B, Resh MD (2000). The phosphatidylinositol polyphosphate 5-phosphatase SHIP1 associates with the dok1 phosphoprotein in bcr-Abl transformed cells.. Cell Signal.

[40] Tamir I, Stolpa JC, Helgason CD, Nakamura K, Bruhns P (2000). The RasGAP-binding protein p62dok is a mediator of inhibitory FcgammaRIIB signals in B cells.. Immunity.

[41] Van den Bergh G, Arckens L (2004). Fluorescent two-dimensional difference gel electrophoresis unveils the potential of gel-based proteomics.. Curr Opin Biotechnol.

[42] Allen LA, Allgood JA, Han X, Wittine LM (2005). Phosphoinositide3-kinase regulates actin polymerization during delayed phagocytosis of Helicobacter pylori.. J Leukoc Biol.

[43] Koch A, Mancini A, El Bounkari O, Tamura T (2005). The SH2-domian-containing inositol 5-phosphatase (SHIP)-2 binds to c-Met directly via tyrosine residue 1356 and involves hepatocyte growth factor (HGF)-induced lamellipodium formation, cell scattering and cell spreading.. Oncogene.

[44] Kamen LA, Levinsohn J, Swanson JA (2007). Differential association of phosphatidylinositol 3-kinase, SHIP-1, and PTEN with forming phagosomes.. Mol Biol Cell.

[45] Ai J, Maturu A, Johnson W, Wang Y, Marsh CB (2006). The inositol phosphatase SHIP-2 down-regulates FcgammaR-mediated phagocytosis in murine macrophages independently of SHIP-1.. Blood.

[46] Crespo P, Schuebel KE, Ostrom AA, Gutkind J, Bustelo XR (1997). Phosphotyrosine-dependent activation of Rac-1 GDP/GTP exchange by the vav proto-oncogene product.. Nature.

[47] Nimmerjahn F, Ravetch JV (2008). Fcgamma receptors as regulators of immune responses.. Nat Rev Immunol.

[48] Nimmerjahn F, Ravetch JV (2007). Antibodie Fc receptors and cancer.. Curr Opin Immunol.

[49] Siberil Dutertre CA, Fridman WH, Teillaud JL (2007). FcgammaR: The key to optimize therapeutic antibodies?. Crit Rev Oncol Hematol.

[50] Parmley SF, Sgarlato GD, Mark J, Prince JB, Remington J (1992). Expression, characterization, and serologic reactivity of recombinant surface antigen P22 of Toxoplasma gondii.. J Clin Microbiol.

[51] Tridandapani S, Wardrop R, Baran CP, Wang Y, Opalek JM (2003). TGF-beta 1 suppresses **[**correction of supresses**]** myeloid Fc gamma receptor function by regulating the expression and function of the common gamma-subunit.. J Immunol.

[52] Eubank TD, Roberts RD, Khan M, Curry JM, Nuovo GJ (2009). Granulocyte macrophage colony-stimulating factor inhibits breast cancer growth and metastasis by invoking an anti-angiogenic program in tumor-educated macrophages.. Cancer Res.

